# Age-related survival gaps in triple-negative breast cancer: a 10-year real-world analysis

**DOI:** 10.1007/s40520-026-03451-y

**Published:** 2026-07-24

**Authors:** Dinis Galhardo, Bárbara Peleteiro, Fernando Osório

**Affiliations:** 1 Faculty of Medicine, University of Porto, Alameda Prof. Hernâni Monteiro, Porto, 4200-319 Portugal; 2 Hospital Epidemiology Center, São João University Hospital, Alameda Prof. Hernâni Monteiro, Porto, 4200-319 Portugal; 3 EPIUnit ITR, Institute of Public Health, University of Porto, Rua das Taipas 135, Porto, 4050-600 Portugal; 4 Department of Public Health and Forensic Sciences, and Medical Education, Faculty of Medicine, University of Porto, Alameda Prof. Hernâni Monteiro, Porto, 4200-319 Portugal; 5 Breast Center, São João University Hospital, Alameda Prof. Hernâni Monteiro, Porto, 4200-319 Portugal; 6 Department of Surgery and Physiology, Faculty of Medicine, University of Porto, Alameda Prof. Hernâni Monteiro, Porto, 4200-319 Portugal; 7 RISE-Health, Faculty of Medicine, University of Porto, Alameda Prof. Hernâni Monteiro, Porto, 4200-319 Portugal

**Keywords:** Aged, Triple negative breast neoplasms, Standard of care, Undertreatment, Ageism, Geriatric assessment

## Abstract

Triple-negative breast cancer (TNBC) is characterized by aggressive biological behavior and poor prognosis. While multimodal treatment is standard, it is frequently withheld from older adults. We evaluated age-related gaps in diagnosis, treatment, and survival, testing whether disparities in clinical management drive worse outcomes in older adults. This 10-year retrospective cohort study (2010–2019) included 289 TNBC patients stratified into three age groups (≤ 45, 46–69 and ≥ 70 years). Clinicopathological features, treatment patterns, adherence to the ESMO-defined standard of care (SOC), and 5-year survival were analyzed. TNBC incidence (10.8%) remained consistent across age groups (*p =* 0.374). Patients aged ≥ 70 years presented with larger tumors (median: 27 mm; *p =* 0.003) and received less intensive diagnostic workups (*p <* 0.001). SOC adherence was significantly lower in older adults (50.9% vs. 85.9% in those ≤ 45 years; *p <* 0.001). SOC nonadherence increased the risk for both overall mortality (OM) (HR: 3.47) and breast cancer-specific mortality (BCSM) (sHR: 2.88) (*p <* 0.001). Failure to achieve pathologic complete response (pCR) tripled mortality risk (OM HR: 3.74, *p* = 0.016; BCSM sHR: 3.54, *p* = 0.018). Older women had lower survival rates than did those aged 46–69 years for both 5-year overall (56.0% vs. 83.0%; *p <* 0.001) and breast cancer-specific survival (63.0% vs. 85.0%; *p =* 0.006). The poorer prognosis in older adults with TNBC is significantly associated with clinical management disparities and low SOC adherence, while histopathological markers of tumor aggressiveness remain highly prevalent across the age spectrum. Daily practice must transition from chronological age-based decisions toward biological fitness to overcome ageism, avoid undertreatment, and ensure personalized therapeutic decision-making.

**Supplementary Information:**

The online version contains supplementary material available at https://doi.org/10.1007/s40520-026-03451-y.

## Introduction

Breast cancer (BC) remains the leading cause of cancer-related mortality among women worldwide. This trend represents an escalating public health challenge in our current aging population, particularly as age is a nonmodifiable risk factor [1, 2]. Among BC molecular subtypes, triple-negative breast cancer (TNBC) is distinguished by its aggressive biological behavior and poor prognosis. Owing to the absence of estrogen and progesterone receptors and the absence of ERBB2 amplification, TNBC lacks targeted endocrine or anti-HER2 therapies [3–5]. Consequently, chemotherapy remains the cornerstone of systemic treatment, complemented more recently by immunotherapy [6]. Owing to its typically high proliferative index and early metastatic potential, a multimodal approach involving surgery, chemotherapy, and radiotherapy is essential to achieve better outcomes [7, 8].

TNBC is traditionally characterized as a disease of younger premenopausal women [3, 9]. Consequently, the disease burden of this molecular subtype in older adults is often undervalued, a disparity reflected in their well-known underrepresentation in clinical trials [10]. Despite proven survival benefits, real-world data suggest that recommended therapies are routinely withheld from older adults [7, 11–14]. This potential undertreatment is difficult to define in older adults [15] and is frequently driven by concerns regarding limiting comorbidities, polypharmacy, and a perceived lower tolerance for treatment-related toxicity [9, 16]. Nevertheless, emerging evidence indicates that chronological age alone is a poor proxy for biological fitness and should not justify the omission of established therapeutic standards [17, 18]. Therefore, to avoid ageism, the integration of a comprehensive geriatric assessment (CGA) is essential for informed and personalized clinical decision-making within a multidisciplinary team [17–19].

Real-world data addressing the impact of adherence to the standard of care (SOC) on the survival of older adults with TNBC are scarce [20]. Most studies focus either on younger cohorts or general breast cancer populations, leading to a knowledge gap regarding how clinical management influences prognosis in older adults [21]. By analyzing over a decade of clinical practice at a reference center, this study seeks to bridge these gaps, providing evidence on how treatment patterns and SOC adherence translate into survival and mortality discrepancies.

In this study, our primary objective was to characterize the age-related differences in diagnostic and treatment approaches. Second, we aimed to determine whether these differences were associated with survival discrepancies across age groups, evaluating the hypothesis that undertreatment is a determinant of worse outcomes in older adults with TNBC.

## Methods

### Study design

This single-center, retrospective cohort study was conducted at the Breast Center of *Unidade Local de Saúde de São João* (BC-ULSSJ) in Porto, Portugal. The study included TNBC patients treated between January 1st, 2010, and December 31st, 2019, and followed until December 31st, 2024. The study was approved by the local institutional ethics committee (CE-273-2025). The STROBE reporting guidelines and checklists are included in Supplement A [22].

### Study population

**Fig. 1:**
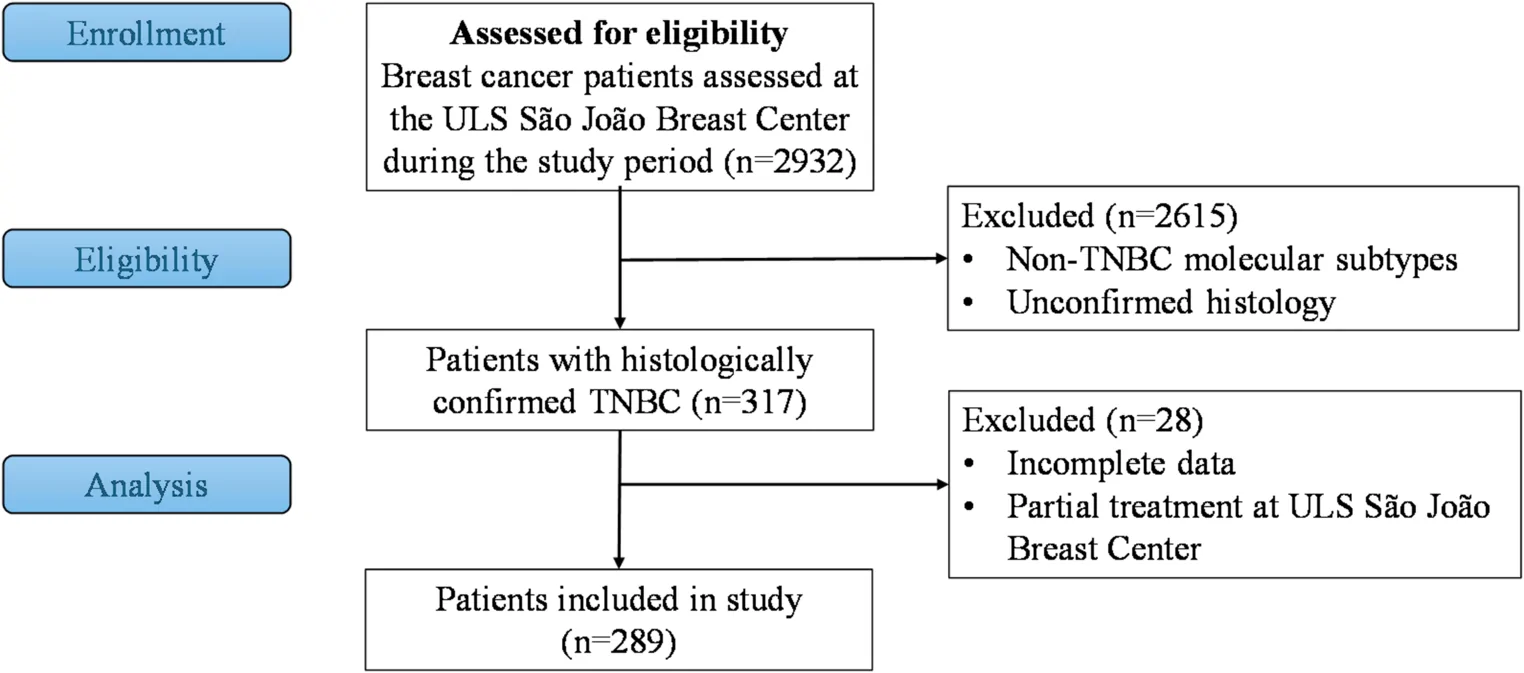
Study flowchart describing the patient selection process. From a total of 2932 breast cancer patients assessed, 2615 were excluded due to non-TNBC histology. Of the 317 confirmed TNBC cases, a further 28 were excluded due to missing data or incomplete treatment at the center, resulting in a final cohort of 289 patients. TNBC: Triple-Negative Breast Cancer

Among the initial cohort of 2,932 BC patients, 317 were identified as having histologically confirmed TNBC (Fig. 1). The eligibility criteria required complete clinical and pathological data, with both diagnosis and treatment performed at the BC-ULSSJ during the study period. Consequently, 28 patients were excluded because of incomplete records or partial treatment at our center, resulting in a final cohort of 289 patients, all of whom were women. Age groups were stratified into ≤ 45, 46–69, and ≥ 70 years according to the age thresholds of the national population-based screening program.

### Data collection

Clinical data were extracted from the electronic medical records. The variables included demographics and clinical staging (cTNM), as well as pathological staging (pTNM) and postneoadjuvant pathological staging (ypTNM), all according to the AJCC 7th edition [23]. Patients with cTNM stage 0 disease were diagnosed with TNBC only after surgery. Invasive tumor grade was assessed using the Bloom-Richardson grading [24].

Adherence to the standard of care (SOC) was defined in accordance with the 2024 ESMO guidelines [8]. For this specific parameter, stage IV patients (*N* = 10) were excluded because of the more individualized nature of therapeutic strategies. A strict binary classification (“adherence” vs. “nonadherence”) was implemented, focusing on the recommended stage-specific systemic treatment sequencing pathways (initial choice and required subsequent systemic continuum). The exact operational criteria, sequencing rules, and deviation definitions utilized for this compliance audit are detailed in Table S1 (Supplement B).

Treatment modalities and outcomes were documented for the entire cohort. However, surgical variables, including type of surgery, residual tumor size, surgical margins (R0), pTNM, and ypTNM were only assessed for patients who underwent surgery. Similarly, the type of chemotherapy was only recorded for the subset of patients who received chemotherapy. Pathologic complete response (pCR) was defined as the absence of residual invasive tumor (0.0 mm) and R0 margins. Survival outcomes and causes of death (BC-related or due to other causes) were documented for the entire cohort and cross-referenced integrating hospital medical records and death certificates obtained from the Portuguese national death registry.

### Statistical analysis

Baseline clinical and pathological characteristics were summarized via descriptive statistics. Continuous variables are presented as the means and standard deviations or the medians and interquartile ranges, as appropriate, whereas categorical variables re expressed as absolute and relative percentages. Comparisons between age groups were performed via the chi-square test or Fisher’s exact test for categorical variables and the Student’s t test or Kruskal-Wallis test for continuous variables.

To evaluate treatment-specific outcomes, the cohort was stratified into two subgroups according to the initial therapeutic approach: neoadjuvant chemotherapy (NAC) or upfront surgery. Survival outcomes, 5-year overall survival (OS), 5-year breast cancer-specific survival (BCSS), and 5-year disease-free survival (DFS), were estimated via the Kaplan–Meier method, with differences between groups assessed via the log-rank (Mantel‒Cox) test. Cox proportional hazards regression was applied to identify prognostic factors associated with overall mortality (OM), with the results reported as hazard ratios (HRs) and 95% confidence intervals (CIs). To accurately analyze breast cancer-specific mortality (BCSM) while accounting for non-cancer deaths as competing risks, a univariable Fine–Gray competing-risks regression model was implemented to calculate subdistribution hazard ratios (sHRs) and 95% confidence intervals (CIs). Additionally, to control for baseline disease severity, a targeted multivariable adjustment for clinical stage (cTNM) was performed using multivariable Cox and Fine–Gray regressions. Due to the reduced number of patients in the ≥ 70 years group, HR calculation for cTNM and pCR was not feasible in this specific subset.

Statistical significance was set at *p <* 0.05, and analyses were conducted via IBM SPSS Statistics (version 30.0) and Stata Statistical Software (version 15).

## Results

Among the 2,932 BC patients, 10.8% were classified as having TNBC. This incidence did not differ significantly across age groups (≤ 45 years: 12.3%; 45–69 years: 10.7%; ≥70 years: 9.9%; *p =* 0.374). The mean age at diagnosis of the 289 patients included in the study was 56.4 ± 14.2 years (range: 28‒93 years). The detailed clinicopathological characteristics are described in Table 1.

**Table 1:** Cohort diagnostic characteristics stratified by age group.

Characteristic	*N*	≤ 45 y, *N* = 68	46–69 y, *N* = 164	≥ 70 y, *N* = 57	*p*-Value
Referral from screening program	289	0 (0.0%)	25 (15.2%)	1 (1.8%)	< 0.001
Clinically palpable mass	282	65 (98.5%)	136 (84.0%)	50 (92.6%)	0.007
Median tumor size by imaging or physical examination [mm. (IQR)]	289	25.0 (20.0**–**35.0)	20.0 (15.0**–**27.0)	27.0 (15.0**–**31.0)	0.003
Magnetic resonance imaging (MRI)	289	39 (57.4%)	63 (38.4%)	8 (14.0%)	< 0.001
Disease extent by imaging or physical examination Localized Multicentric/Multifocal	289	- 59 (86.8%) 9 (13.2%)	- 145 (89.0%) 18 (11.0%)	- 50 (87.7%) 7 (12.3%)	0.880
Genetic testing No genetic assessment BRCA1 BRCA2	289	- 13 (19.1%) 7 (10.3%) 1 (1.5%)	- 85 (51.8%) 5 (3.0%) 3 (1.8%)	- 52 (91.2%) 0 (0.0%) 1 (1.8%)	< 0.001
AJCC clinical staging (cTNM) 0 I II III IV	289	- 0 (0.0%) 19 (27.9%) 40 (58.8%) 5 (7.4%) 4 (5.9%)	- 3 (1.8%) 72 (43.9%) 81 (49.4%) 4 (2.4%) 4 (2.4%)	- 0 (0.0%) 16 (28.1%) 35 (61.4%) 4 (7.0%) 2 (3.5%)	0.085
ESMO-defined adherence to the standard of care (SOC)	279	55 (85.9%)	125 (78.1%)	28 (50.9%)	< 0.001
First treatment Neoadjuvant chemotherapy Surgery Best supportive care	289	- 46 (67.6%) 22 (32.4%) 0 (0.0%)	- 73 (44.5%) 90 (54.9%) 1 (0.6%)	- 13 (22.8%) 42 (73.7%) 2 (3.5%)	< 0.001

With respect to detection and diagnosis, 15.2% of women aged 46–69 years were referred from population-based screening, compared with 1.8% in the older group and none in the younger group (*p <* 0.001). A clinically palpable mass was significantly more common in the extreme groups than in the 46–69 year group (*p =* 0.007). Smaller tumors were observed in women aged 46–69 years (median: 20 mm), compared with those aged ≤ 45 years (median: 25 mm) and ≥ 70 years (median: 27 mm; *p =* 0.003). While the distribution of the cTNM suggested a similar pattern of earlier detection in women aged 46–69 years, but the difference was not statistically significant (*p =* 0.085), individual analysis of the clinical tumor (cT) stage revealed a significant difference (*p =* 0.008). Furthermore, the use of magnetic resonance imaging (MRI) was significantly greater in younger women (57.4%) than in those aged 46–69 years (38.4%), and this difference was more pronounced in older women (14.0%; *p <* 0.001).

Women aged ≥ 70 years underwent genetic assessment less frequently (8.8%) compared with those aged ≤ 45 years (80.9%) and 46–69 years (48.2%; *p <* 0.001). *BRCA1* mutations were identified in 10.3% of women aged ≤ 45 years and 3.0% of those aged 46–69 years, whereas no *BRCA1* mutations were detected in those aged ≥ 70 years. However, *BRCA2* mutation positivity was similar across age groups (1.5–1.8%).

The highest ESMO-defined SOC compliance was observed in women aged ≤ 45 years (85.9%) and in those aged 46–69 years (78.1%), whereas the lowest was 50.9% in older adults (*p <* 0.001). Upfront surgery was the predominant treatment choice for older women (73.7% vs. 22.8% NAC). In contrast, NAC was the preferred initial approach for younger patients (67.6% vs. 32.4% upfront surgery; *p <* 0.001). Due to severe comorbidities, 3 patients received best supportive care.

In the analysis of the upfront surgery subgroup (Table 2), patients aged ≥ 70 years presented with a greater proportion of stage II disease (66.7%) than did to those aged ≤ 45 and 46–69 years, whereas stage I disease predominated (63.6% and 60.0%, respectively; *p =* 0.003). Consistent with these findings, pathological tumor size was significantly greater in patients aged ≥ 70 years (median: 22.5 mm) than in younger adults (*p =* 0.008). In this subgroup, adjuvant chemotherapy (*p <* 0.001) and radiotherapy (*p =* 0.012) were significantly less common in women aged ≥ 70 years.

**Table 2:** Cohort clinicopathological characteristics divided into two subgroups according to the first treatment performed and stratified by age group.

Characteristic	*N*	≤ 45 y, *N* = 46	46–69 y, *N* = 73	≥ 70 y, *N* = 13	*p*-Value
*Neoadjuvant chemotherapy (NAC) (N = 132)*					
AJCC clinical staging (cTNM) I II III IV	132	- 5 (10.9%) 34 (73.9%) 5 (10.9%) 2 (4.3%)	- 18 (24.7%) 51 (69.9%) 2 (2.7%) 2 (2.7%)	- 3 (23.1%) 6 (46.2%) 3 (23.1%) 1 (7.7%)	0.033
Type of neoadjuvant chemotherapy Anthracyclines with taxanes Taxanes without anthracyclines Other	132	- 38 (82.6%) 4 (8.7%) 4 (8.7%)	- 51 (69.9%) 11 (15.1%) 11 (15.1%)	- 3 (23.1%) 19 (76.9%) 0 (0.0%)	< 0.001
Type of breast surgery Breast-conserving surgery Mastectomy With immediate breast reconstruction	122 46	- 22 (51.2%) 21 (48.8%) 6 (28.6%)	- 48 (70.6%) 20 (29.4%) 4 (20.0%)	- 6 (54.5%) 5 (45.5%) 0 (0.0%)	0.103
No surgery	132	3 (6.5%)	5 (6.8%)	2 (15.4%)	0.533
Surgical staging of the axilla Sentinel lymph node biopsy (SLNB) Axillary lymph node dissection (ALND) SLNB plus ALND None	132	- 31 (67.4%) 5 (10.9%) 7 (15.2%) 3 (6.5%)	- 44 (60.3%) 9 (12.3%) 15 (20.5%) 5 (6.8%)	- 6 (46.2%) 3 (23.1%) 2 (15.4%) 2 (15.4%)	0.723
Final Pathology No tumor/Pathologic complete response (pCR) Residual invasive tumor	122	- 22 (51.2%) 21 (48.8%)	- 24 (35.3%) 44 (64.7%)	- 1 (9.1%) 10 (90.9%)	0.025
Median residual tumor size [mm. (IQR)]	122	0.0 (0.0**–**22.0)	7.5 (0.0**–**17.8)	15.0 (5.0**–**22.0)	0.176
Clear margins (R0)	122	43 (100%)	65 (95.6%)	9 (90.9%)	0.234
AJCC post-neoadjuvant pathological staging (ypTNM) 0 I II III	122	- 22 (51.2%) 6 (14.0%) 9 (20.9%) 6 (14.0%)	- 24 (35.3%) 23 (33.8%) 16 (23.5%) 5 (7.4%)	- 1 (9.1%) 3 (27.3%) 6 (54.5%) 1 (9.1%)	0.033
Adjuvant radiotherapy	132	31 (67.4%)	53 (72.6%)	6 (46.2%)	0.167
Adjuvant chemotherapy	132	5 (10.9%)	5 (6.8%)	1 (7.7%)	0.739
Type of adjuvant chemotherapy Anthracyclines with taxanes Taxanes without anthracyclines Capecitabine	11	- 1 (20.0%) 2 (40.0%) 2 (40.0%)	- 1 (20.0%) 0 (0.0%) 4 (80.0%)	- 0 (0.0%) 1 (100.0%) 0 (0.0%)	0.274
*Upfront surgery* (*N = 154)*					
AJCC clinical staging (cTNM) 0 I II III IV	154	- 0 (0.0%) 14 (63.6%) 6 (27.3%) 0 (0.0%) 2 (9.1%)	- 3 (3.3%) 54 (60.0%) 30 (33.3%) 2 (2.2%) 1 (1.1%)	- 0 (0.0%) 13 (31.0%) 35 (66.7%) 1 (2.4%) 0 (0.0%)	0.003
Type of breast surgery Breast-conserving surgery Mastectomy With immediate breast reconstruction (IBR)	154	- 17 (77.3%) 5 (22.7%) 1 (20.0%)	- 64 (71.1%) 26 (28.9%) 6 (23.1%)	- 23 (54.8%) 19 (45.2%) 0 (0.0%)	0.100
Surgical staging of the axilla Sentinel lymph node biopsy (SLNB) Axillary lymph node dissection (ALND) SLNB plus ALND None	154	- 18 (81.8%) 0 (0.0%) 4 (18.2%) 0 (0.0%)	- 68 (75.6%) 0 (0.0%) 20 (22.2%) 2 (2.2%)	- 24 (57.1%) 1 (2.4%) 13 (31.0%) 4 (9.5%)	0.101
Median pathological tumor size [mm. (IQR)]	154	17.5 (13.0**–**24.3)	16.0 (11.0**–**24.0)	22.5 (16.0**–**30.0)	0.008
Clear margins (R0)	154	22 (100.0%)	82 (91.1%)	39 (92.2%)	0.349
AJCC pathological staging (pTNM) I II III	154	- 12 (54.5%) 18 (40.9%) 7 (4.5%)	- 45 (50.0%) 36 (40.0%) 9 (10.0%)	- 10 (28.6%) 23 (54.8%) 7 (16.7%)	0.131
Adjuvant radiotherapy	154	19 (86.4%)	61 (67.8%)	21 (50.0%)	0.012
Adjuvant chemotherapy	154	21 (95.5%)	78 (86.7%)	21 (50.0%)	< 0.001
Type of adjuvant chemotherapy Anthracyclines with taxanes Taxanes without anthracyclines Other	120	- 19 (90.4%) 1 (4.8%) 1 (4.8%)	- 62 (79.5%) 56 (17.9%) 2 (2.6%)	- 2 (9.5%) 16 (76.2%) 3 (14.3%)	< 0.001

In the NAC subgroup (Table 2), pathologic complete response (pCR) rates varied significantly between age groups: 51.2% in younger women, 35.3% in women aged 46–69 years, and 9.1% in older women (*p =* 0.025). This trend was reflected in the median residual tumor size: 0.0 mm in patients aged ≤ 45 years, 7.5 mm in those aged 46–69 years, and 15.0 mm in those aged ≥ 70 years, although the difference was not statistically significant. Nevertheless, the distribution of ypTNM differed significantly between groups (*p =* 0.033), indicating a predominance of stages 0/I in the ≤ 45 years group compared with stages II/III in the ≥ 70 years group.

Older adults, in both subgroups, had higher rates of mastectomies, all without immediate breast reconstruction (IBR), and were less likely to receive anthracycline-based chemotherapy regimens (*p <* 0.001). The majority of the tumors were no special type (NST) invasive carcinoma (69.8%; *p =* 0.145) or grade 3 (80.9%; *p =* 0.123). In addition, the overall proportion of multicentric tumors was 11.7% (*p =* 0.880), and that of lymphovascular invasion (LVI) was 21.9% (*p =* 0.323).

**Fig. 2:**
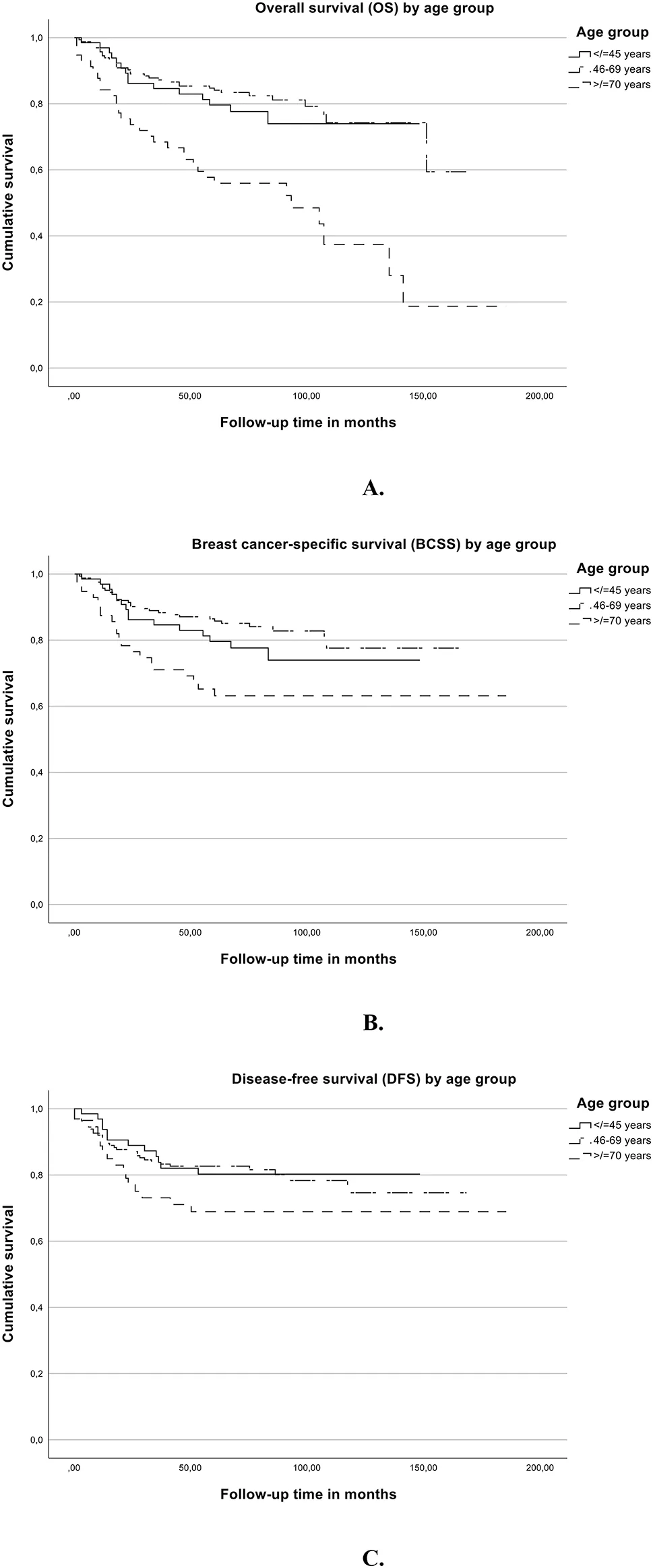
Survival curves stratified by age group (≤ 45, 46–69, and ≥ 70 years). Panels display: (**A**) overall survival, (**B**) breast cancer-specific survival, and (**C**) disease-free survival. Follow-up time is represented in months

Significant differences in overall survival (OS) and breast cancer-specific survival (BCSS) were observed (*p <* 0.001 and *p =* 0.006, respectively), as detailed in Table 3 and in Fig. 2, with older adults exhibiting the lowest survival rates (OS: 56.0%; BCSS: 63.0%). Although not statistically significant, worse outcomes in older adults were also observed in terms of disease-free survival (DFS) and recurrence rates (*p =* 0.209).

Patients aged ≥ 70 years presented an increased risk of overall mortality (OM) (HR: 3.22; 95% CI: 1.97–5.26; *p <* 0.001) and breast cancer-specific mortality (BCSM) (HR: 2.50; 95% CI: 1.41–4.34; *p =* 0.002) compared with the reference group (46–69 years). As detailed in Table 4, cTNM stage III disease was associated with higher OM (HR: 16.50, 95% CI: 6.57–41.46, *p <* 0.001) and BCSM (sHR: 14.86; 95% CI: 5.21–43.34; *p <* 0.001) than stage I disease (the reference group), a pattern also observed in stage IV disease.

**Table 3:** Cohort prognostic characteristics stratified by age group.

Characteristic	*N*	≤ 45 y, *N* = 68	46–69 y, *N* = 164	≥ 70 y, *N* = 57	*p*-Value
5-year overall survival (OS)	289	78.0%	83.0%	56.0%	< 0.001
Vital status at follow-up Alive Deceased	289	- 53 (77.9%) 15 (22.1%)	- 131 (79.9%) 33 (20.1%)	- 26 (45.6%) 31 (54.4%)	< 0.001
5-year breast cancer specific survival (BCSS)	289	78.0%	85.0%	63.0%	0.006
Cause of death With/from breast cancer Other causes Missing data	79	- 15 (100%) 0 (0.0%) 0 (0.0%)	- 28 (84.8%) 5 (15.2%) 0 (0.0%)	- 20 (64.5%) 9 (29.0%) 2 (9.7%)	0.042
5-year disease free survival (DFS)	289	80.0%	83.0%	69.0%	0.209
Any recurrence	289	12 (17.6%)	32 (19.5%)	16 (28.1%)	0.300
Recurrence Local Regional At distance	289	- 5 (7.4%) 3 (4.4%) 11 (16.2%)	- 11 (6.7%) 6 (3.7%) 24 (14.6%)	- 7 (12.3%) 6 (10.5%) 14 (24.6%)	- 0.399 0.125 0.223

The first treatment approach (upfront surgery vs. NAC) did not significantly impact mortality. However, SOC nonadherence was associated with a more than threefold increased risk for OM (HR: 3.47; 95% CI: 2.16–5.57; *p <* 0.001) and a significant increase for BCSM (sHR: 2.88; 95% CI: 1.67–4.95; *p <* 0.001). Furthermore, the presence of a residual tumor (nonpCR) after NAC was associated with an unfavorable prognosis for OM (HR: 3.74; 95% CI: 1.28–10.91; *p =* 0.016) and BCSM (sHR: 3.54; 95% CI: 1.24–10.10; *p =* 0.018). The cTNM-adjusted HR for SOC nonadherence was 3.53 (95% CI: 2.19–5.70; *p <* 0.001) for OM, and the adjusted sHR was 2.63 (95% CI: 1.55–4.46; *p <* 0.001) for BCSM.

**Table 4:** Cohort overall and breast cancer-specific mortality characteristics stratified by age group.

Characteristic	*N*	Overall mortality (OM)				Breast cancer–specific mortality (BCSM)			
		Total	≤ 45 y	46–69 y	≥ 70 y	Total	≤ 45 y	46–69 y	≥ 70 y
cTNM I II III IV	289	- 1 [ref] 4.37 (2.16–8.70) *p* < 0.001 16.50 (6.57–41.46) *p* < 0.001 34.92 (14.09–86.56) *p* < 0.001	- 1 [ref] 3.64 (0.46–29.11) *p* = 0.223 11.65 (1.05–129.03) *p* = 0.045 29.98 (2.99–243.77) *p* = 0.003	- 1 [ref] 3.34 (1.32–8.46) *p* = 0.011 25.30 (6.84–93.55) *p* < 0.001 49.25 (12.83–187.61) *p* < 0.001	- 1 [ref] 6.17 (1.82–20.90) *p* = 0.003 10.60 (2.07–53.44) *p* = 0.005 91.45 (11.66–717.45) *p* < 0.001	- 1 [ref] 5.12 (2.19–11.96) *p* < 0.001 14.86 (5.21–43.34) *p* < 0.001 46.80 (18.69–117.22) *p* < 0.001	- 1 [ref] 2.86 (1.05–7.83) *p* = 0.040 27.39 (10.35–72.47) *p* < 0.001 54.95 (16.30–185.18) *p* < 0.001	- 1 [ref] 3.64 (0.47–28.12) *p* = 0.216 11.65 (1.07–127.17) *p* = 0.044 26.98 (3.84–189.53) *p* = 0.001	- – – – –
First treatment NAC Surgery	289	- 1 [ref] 0.89 (0.56–1.43) *p* = 0.636	- 1 [ref] 0.71 (0.23–2.24) *p* = 0.561	- 1 [ref] 0.62 (0.30–1.27) *p* = 0.188	- 1 [ref] 0.90 (0.36–2.26) *p* = 0.822	- 1 [ref] 0.78 (0.47–1.29) *p* = 0.329	- 1 [ref] 0.53 (0.25–1.16) *p* = 0.113	- 1 [ref] 0.71 (0.24–2.08) *p* = 0.534	- 1 [ref] 1.11 (0.37–3.33) *p* = 0.857
SOC No SOC	279	1 [ref] 3.47 (2.16–5.57) *p* < 0.001	1 [ref] 5.00 (1.45–17.28) *p* = 0.011	1 [ref] 2.90 (1.38–6.08) *p* = 0.005	1 [ref] 2.16 (1.02–4.55) *p* = 0.044	1 [ref] 2.88 (1.67–4.95) *p* < 0.001	1 [ref] 2.34 (1.01–5.40) *p* = 0.047	1 [ref] 5.00 (1.43–17.47) *p* = 0.012	1 [ref] 1.89 (0.75–4.78) *p* = 0.179
pCR Residual tumor	122	1 [ref] 3.74 (1.28–10.91) *p* = 0.016	1 [ref] 10.86 (1.36–86.95) *p* = 0.025	1 [ref] 1.69 (0.46–6.27) *p* = 0.429	– –	1 [ref] 3.54 (1.24–10.10) *p* = 0.018	1 [ref] 10.86 (1.43–82.68) *p* = 0.021	1 [ref] 1.69 (0.47–6.05) *p* = 0.417	– –

## Discussion

Our study challenges the traditional view that TNBC is a molecular subtype affecting predominantly premenopausal women [3, 7, 20]. Our real-world data show that the incidence and histopathological markers remain consistent across age groups, making its absolute burden an escalating public health challenge [9, 13], especially as longevity among women continues to rise.

Despite a uniform incidence, major disparities were observed in the diagnostic landscape, particularly regarding tumor burden at presentation. Compared with the extreme age groups, the 46–69 group presented with nonpalpable, smaller tumors. This is likely attributable to the breast cancer screening program in Portugal, which, until December 2024, targeted women aged 50–69 years. Our results align with those previously reported in a similar Portuguese cohort [20], identifying a clear association between screening age limits and more advanced clinical stages at diagnosis. Furthermore, international studies and guidelines [7, 16] corroborate that older adults, who fall outside screening intervals, are more likely to be diagnosed through clinical symptoms rather than imaging, inevitably leading to a later clinical stage at presentation.

We also observed less locoregional MRI staging in older adults, likely influenced by the higher sensitivity of mammography in the typically less dense breasts of older women [25], leading to a clinical perception that further imaging is redundant. However, this omission may be clinically detrimental, as MRI remains the most sensitive modality for detecting multifocality and multicentricity [25]. Given that ESMO guidelines [8] define accurate assessment of disease extent as a prerequisite for optimal surgical planning, forgoing MRI increases the risk of underestimating the true tumor burden, potentially compromising therapeutic decisions and outcomes such as the achievement of R0 resection margins.

Similarly, genetic assessment was markedly underutilized in the older group. This is particularly striking, given that current international guidelines firmly recommend genetic testing for TNBC patients regardless of age, owing to the high prevalence of germline *BRCA* mutations in this molecular subtype [3, 8]. In the current era of precision medicine, this disparity represents a critical gap in treatment optimization, as these mutations guide the use of targeted therapies (e.g., PARP inhibitors), which have demonstrated substantial survival benefits in the adjuvant setting [26]. By denying that older adults have access to such assessments, we are excluding them from targeted therapies, while also precluding essential familial risk stratification.

Notably, neoadjuvant immunotherapy was not administered during the study period, as its implementation at BC-ULSSJ only began in 2023 [6]. Nonetheless, while international guidelines favored NAC as the first-line SOC treatment for TNBC during our study timeline [8], older adults were less likely to receive it, which is consistent with previous research [7]. Given that older women are diagnosed with more advanced tumors for which NAC is the recommended initial strategy, it is even less reasonable to begin treatment with surgery. This preference for upfront surgery is driven by chronological age rather than tumor stage and reflects a persistent gap between guidelines and real-world practice [13, 16].

This potential undertreatment is challenging to define in older adults without a CGA [15]. Nonetheless, the preference for anthracycline-free, taxane-based regimens over anthracycline-based combinations suggests that clinicians may prioritize cardiac safety over oncological efficacy [27, 28]. This trend is frequently reported in older adults, where concerns regarding comorbidities and polypharmacy often lead to the attenuation of chemotherapy intensity [4, 9, 16]. This reduction in intensity is likely reflected in the pCR rates: while approximately half of the younger group achieved pCR, fewer than one in ten older women achieved a complete response, which is consistent with other real-world studies [7, 12]. Given that the pCR rate is a robust surrogate marker for survival [3, 8], this marked difference in chemosensitivity potentially mirrors the outcome disparities found in our study.

With respect to the upfront surgery subgroup, nearly all younger patients received adjuvant chemotherapy and radiotherapy, whereas both rates decreased to half in older adults. This represents a marked deviation from multimodal treatment strategies [5] and aligns with recent studies [11, 12]. Furthermore, older women had higher mastectomy rates, and IBR was entirely absent. This absolute disparity contrasts with that of the younger group and contradicts the EUSOMA/SIOG recommendations [16], which emphasize that age alone should not preclude breast-conserving strategies or body image self-esteem.

This cumulative deviation from multimodal strategies is best captured by ESMO-defined SOC adherence [8]. While these guidelines were followed in most younger patients, adherence decreased to half in the older group, reinforcing that chronological age acts as a decisive, yet often unjustified, barrier to SOC [13]. In the absence of documented patient preferences or a formal CGA implementation, the decision-making process appears to prioritize chronological age over the EUSOMA/SIOG guidelines [8, 16] for half of the elderly population.

The mortality analysis revealed that the observed survival disparity is not merely a consequence of chronological aging, but rather a reflection of the intensity of the treatment administered. As anticipated, the cTNM emerged as a predictor of prognosis [23]. Similarly, nonadherence to SOC protocols was also associated with worse outcomes, tripling the risk for both OM and BCSM. Interestingly, starting with NAC or with upfront surgery did not impact mortality, suggesting that the survival disparities between age groups are not a byproduct of surgical timing but rather a consequence of the subsequent omission of SOC, which is consistent with prior analyses [11, 21]. Furthermore, the scarcity of older patients who achieved pCR, a phenomenon already reported [14], precluded a reliable statistical assessment of group-specific HRs for this parameter. Nonetheless, in the overall population, the failure to achieve pCR resulted in a threefold increase in the BCSM risk, underscoring the prognostic value of an optimal therapeutic response [3, 8].

This study has several strengths, notably the provision of robust real-world data from an EUSOMA-certified center, reflecting clinical practice beyond the controlled environment of clinical trials, alongside a 10-year longitudinal series, with a minimum 5-year follow-up. The three-age-group stratification (≤ 45, 46–69, and ≥ 70 years) represents a key methodological asset, allowing for a nuanced evaluation of how chronological age individually influences the diagnostic and therapeutic journey, a granularity often lost in studies utilizing binary age cut-offs. Additionally, a distinct methodological advantage is the implementation of a univariable Fine–Gray competing-risks analysis to control for non-cancer mortality, alongside a targeted multivariable adjustment for clinical stage (cTNM). The consistency between the univariable and adjusted estimates indicates that deviation from standard systemic therapeutic pathways carries independent prognostic weight.

However, certain limitations must be acknowledged, including the retrospective, single-center design, which may limit generalizability and carry inherent risks of selection bias. Furthermore, the lack of a formal CGA and complete records on patient comorbidities, preferences, and potential clinical contraindications precluded a direct association between frailty and lower treatment intensity. Consequently, because the SOC compliance metric was restricted to a strict binary classification focused on systemic sequencing pathways, this cumulative non-adherence group represents an aggregate of all sequencing deviations, preventing differentiation between clinically justified variations and unjustified therapeutic omissions. Finally, conditional local therapeutic modalities, such as radiotherapy, were not factored into this compliance framework.

Chronological age should no longer serve as a proxy for frailty or as a justification for withholding current best practices. To address these disparities, clinicians must transition from chronological age to biological fitness, incorporating standardized geriatric tools to tailor individual therapeutic strategies accurately [16, 19]. The active involvement of geriatricians is indispensable, as integrating these geriatric assessments not only mitigates treatment-related toxicity but also translates into personalized interventions [17, 18]. This comprehensive approach is essential to ensure that biologically fit older adults with TNBC are not denied curative SOC on the basis of their birth date.

## Conclusion

Our study demonstrated that triple-negative breast cancer is not only a disease of young but also of older adults, as its incidence and histopathological markers remain consistent across the age spectrum. Despite these clinicopathological similarities, our real-world data revealed significant prognosis discrepancies, with older adults experiencing markedly inferior overall survival and breast cancer-specific survival. Hence, these findings indicate that the poorer prognosis observed in older adults is heavily driven by critical disparities in diagnostic and treatment management, alongside their historical underrepresentation in randomized controlled trials.

Ultimately, we highlight an urgent need to move beyond chronological age and transition toward the evaluation of biological fitness in therapeutic decision-making. Integrating comprehensive geriatric assessment into daily clinical practice is essential for implementing equitable, tailored strategies to overcome ageism and close the survival gap identified in our study.

## Supplementary Information

Below is the link to the electronic supplementary material.

Supplementary Material 1

## Data Availability

All research data were archived in the hospital’s database and can be provided, if requested, to the corresponding author.
